# Effect of Collagen Hydrolysates from Silver Carp Skin (*Hypophthalmichthys molitrix*) on Osteoporosis in Chronologically Aged Mice: Increasing Bone Remodeling

**DOI:** 10.3390/nu10101434

**Published:** 2018-10-04

**Authors:** Ling Zhang, Siqi Zhang, Hongdong Song, Bo Li

**Affiliations:** 1 Beijing Advanced Innovation Center for Food Nutrition and Human Health, College of Food Science and Nutritional Engineering, China Agricultural University, Beijing 100083, China; zhanglingys@outlook.com (L.Z.); zsq199312@163.com (S.Z.); songhd@cau.edu.cn (H.S.); 2 Beijing Higher Institution Engineering Research Center of Animal Product, Beijing 100083, China

**Keywords:** osteoporosis, chronologically aged mice, collagen hydrolysates, bone remodeling

## Abstract

Osteoporosis is a common skeletal disorder in humans and gelatin hydrolysates from mammals have been reported to improve osteoporosis. In this study, 13-month-old mice were used to evaluate the effects of collagen hydrolysates (CHs) from silver carp skin on osteoporosis. No significant differences were observed in mice body weight, spleen or thymus indices after daily intake of antioxidant collagen hydrolysates (ACH; 200 mg/kg body weight (bw) (LACH), 400 mg/kg bw (MACH), 800 mg/kg bw (HACH)), collagenase hydrolyzed collagen hydrolysates (CCH) or proline (400 mg/kg body weight) for eight weeks, respectively. ACH tended to improve bone mineral density, increase bone hydroxyproline content, enhance alkaline phosphatase (ALP) level and reduce tartrate-resistant acid phosphatase 5b (TRAP-5b) activity in serum, with significant differences observed between the MACH and model groups (*p* < 0.05). ACH exerted a better effect on osteoporosis than CCH at the identical dose, whereas proline had no significant effect on repairing osteoporosis compared to the model group. Western blotting results demonstrated that CHs mainly increased bone remodeling by stimulating the transforming growth factor β1 (TGF-β1)/Smad signaling pathway and improving the interaction between collagen and α2β1 integrin. The results indicated that CHs from fish could be applied to alleviate osteoporosis or treat bone loss.

## 1. Introduction

The prevalence of osteoporosis is higher in the elderly. Osteoporosis is defined as a chronic and asymptomatic disease characterized by low bone mineral density (BMD) and skeletal microarchitectural deterioration, leading to increased risk of fracture and associated comorbidities, such as lumbago and arthralgia [1,2,3]. The pathogenesis of osteoporosis is mainly due to age-related estrogen drop, genetic factors and lifestyle [4]. Osteoporosis results from the imbalance in bone turnover associated with excessive resorption and exiguous remodeling. Physical activity and nutrition supplementation (especially vitamin D and calcium) are considered practical measures for preventing and treating osteoporosis in elderly populations.

Commercially, medications for osteoporotic therapy are mainly distributed into two categories: anti-resorptive medications and bone-forming (anabolic) medications [5]. Bisphosphonate, the gold standard of anti-catabolic therapy, has been clinically used to treat fractures of osteoporotic syndrome and the hip [6]. Parathyroid hormone (PTH) is one of the approved anabolic candidates used for the treatment of osteoporosis in developed countries [7]. The beneficial anabolic effect of PTH administration on bone in various animal models and humans has been proved [8]. However, it has been emphasized that compliance to such therapy tends to be poor and benefit does not continue after ending treatment [9]. Besides, the long-term application of anti-osteoporotic drugs comes with serious side effects [10]. Therefore, there is an urgent need to find safe and effective alternatives for osteoporosis treatments.

For decades, researchers have mainly focused on how to improve bone mineral density (BMD) in osteoporosis patients. Targeting the collagen metabolism in bone might be a new therapeutic approach to improve skeleton health. The capacity of bone to resist fractures and mechanical forces depends not only on the quantity of bone mineral, but also on the collagen framework [11,12]. As the major structural element in the extracellular matrix of all connective tissues, collagens represent about 80% of the total protein [13]. Collagens form the scaffold for the attachment of cells and the anchorage of macromolecules and minerals [9]. Furthermore, bone collagens can bind and store growth factors and cytokines, such as insulin like growth factor (IGF) I and II, which are released during bone degradation and in turn promote bone formation. Due to the importance of collagen in bone capacity, the metabolism of collagen has received great interest from scientists.

According to the nutritional strategy that providing a sufficiently bioavailable amount of constitutive elements, such as calcium and proteins, leads to effective prevention of bone loss, many studies have reported the effect of collagen hydrolysates (CHs) on improving osteoporosis [9]. In an in vitro study, the collagen hydrolysate-derived Asp-Gly-Glu-Ala peptide was capable of triggering osteoblast metabolism in bone marrow cells. Moreover, Hyp-containing peptides foster osteoblast activity, resulting in increased bone mineralization and synthesis of organic bone components [9]. An in vivo study showed that fish-bone peptides could increase calcium retention and inhibit bone loss in ovariectomised (OVX) rats [14]. Besides, CHs from pig or deer also exerted significant effects on preventing bone loss, improving BMD, and increasing histomorphometric parameters and mechanical indicators in OVX animal models [15,16]. Clinical trials further demonstrated that special collagen peptides improved BMD and bone markers in postmenopausal women [17]. However, previous investigations mainly focused on studying the effect of mammalian or bone CHs on osteoporosis. Furthermore, the changes in collagen metabolism and the effect of CHs on these lack examinations in a head-to head comparison between young and healthy and old and osteoporotic individuals.

Porcine skin, bovine hide and cattle bones are abundant sources for gelation. However, the industrial application of collagen or gelatin from non-mammalian species is gaining more attention due to socio-cultural and sanitary aspects [18]. Hence, the CHs from silver carp (one of the four major cultured species in China) skin were used in the present study. In our previous study, the antioxidant collagen hydrolysates (ACH) with the lowest molecular distribution and the strongest antioxidant activities exerted the best effect on alleviating mice skin photoaging compared to other two CHs with higher molecular weight distribution [19]. This study was further conducted to evaluate the effect of ACH on attenuating osteoporosis or bone loss with young healthy mice as control. Besides, in order to study whether collagenase, an endogenous enzyme responsible for degrading collagen in vivo, can be used as a novel enzyme to produce CHs, the effect collagenase hydrolyzed collagen hydrolysates (CCH) on osteoporosis was also studied. Therefore, in the present study, the preventive effects of two kinds of CHs from silver carp skin on osteoporosis were investigated in a 13-month-old mouse model. Moreover, the mechanism of CHs on bone collagen synthesis was explored by western blotting analysis.

## 2. Materials and Methods

### 2.1. Materials

Bacterial collagenase and alkaline proteinase were purchased from Sigma-Aldrich (St. Louis, MO, USA). The bicinchoninic acid (BCA) protein assay kit was purchased from Beijing Solarbio Science and Technology Co., Ltd. (Beijing, China). Commercial kits used for determining hydroxyproline (Hyp), alkaline phosphatase (ALP) concentration and tartrate-resistant acid phosphatase-5b (TRAP-5b) activity were purchased from Jiancheng Inst. of Biotechnology (Nanjing, China). Rabbit monoclonal anti-transforming growth factor β1 (TGFβ) and rabbit monoclonal anti-Smad3 were purchased from Cell Signaling Technology (Danvers, MA, USA). Rabbit monoclonal anti-Smad7, rabbit monoclonal anti-α2 and rabbit monoclonal anti-β1 were purchased from Abcam (Cambridge, MA, USA). Other chemicals used in this study were of analytical grade or better and were commercially available.

### 2.2. Preparation of Collagen Hydrolysates (CHs)

Gelatin was extracted from silver carp skin according to our previous report [20]. The ACH with low molecular weight distribution was obtained by alkaline proteinase hydrolysis based on our previous method [19]. Gelatin was also hydrolyzed by another enzyme, collagenase, to obtain CCH with a similar molecular weight distribution and antioxidant activities to ACH. After freeze-drying, two kinds of CHs were stored at −80 °C until use.

### 2.3. Molecular Weight (MW) Distribution

The molecular weight distribution of CHs was determined using a Shimadzu LC-15C high performance liquid chromatography (HPLC) system (Shimadzu, Tokyo, Japan) equipped with a TSK gel G2000 SWXL column (7.8 mm × 300 mm, Tosoh, Tokyo, Japan). Samples were loaded onto the HPLC and eluted with 45% (v/v) acetonitrile, containing 0.1% (v/v) trifluoroacetic acid, at a flow rate of 0.5 mL/min and monitored at 214 nm by an ultraviolet detector at room temperature. A molecular weight calibration curve (y = −0.1881x + 6.5867, y: log MW, x: time, R^2^ = 0.9954) was obtained from the average retention times of the following standards: Gly–Ser (146 Da), Asn–Cys–Ser (322 Da), Trp–Pro–Trp–Trp (674 Da), bacitracin (1423 Da) and aprotinin (6512 Da) [19].

### 2.4. Amino Acid Composition

The amino acid compositions of ACH and CCH were analyzed using the phenylthiocarbamyl (PITC) pre-column derivation by a Shimadzu LC-15C HPLC system based on the previous method [21]. Samples were hydrolyzed using 6 M HCl at 110 °C for 24 h. The contents of the individual amino acids were expressed as g per 100 g of the total amino acids in each sample.

### 2.5. Animals, Diets, and Treatments

The animal experiment was carried out according to the standard guidelines for animal care and under the protocols approved by the Welfare Committee of the Centre of Experimental Animal, Beijing, China (Permission number: KY150018). The experiment was performed in the Experimental Animal Centre, Supervision and Testing Centre for Genetically Modified Organisms (GMOs) and Food Safety, Ministry of Agriculture (specific pathogen free (SPF) grade, Beijing, China).

Two-month-old (young mice, 28 ± 2 g, SPF grade) and 13-month-old (old mice, 45 ± 5 g, SPF grade) female Kunming mice were acquired from Sibeifu (Beijing, China) Laboratory Animal Science and Technology Co., Ltd. (Beijing, China). The 13-month-old mice were assigned to 6 groups (*n* = 10/group) based on body weight, including the model group (M) and treatment groups. The model group and the two-month-old young control group (YC) were given 0.2 mL normal saline daily, whereas treatment groups were given 0.2 mL ACH daily at doses of 200 mg/kg body weight (bw) (LACH), 400 mg/kg bw (MACH) and 800 mg/kg bw (HACH) respectively by intragastric administration. Besides, groups administrated with 0.2 mL CCH and proline at the medium dose (400 mg/kg bw) respectively were used as control groups (named MCCH and MP, respectively). Here the medium dose was used for control groups to ensure enough supplements have been offered. All the mice had free access to a controlled diet (AIN-93M, the second of two open formulations published by the American Institute of Nutrition (AIN) committee in 1993 to provide nutrients in concentrations to maintain adult rat or mouse populations) and water. The weight of mice was measured weekly. After 8 weeks, mice were sacrificed and samples were collected for further analysis. The spleen and thymus were excised from mice and weighed immediately. The spleen index (SI) and thymus index (TI) were calculated as indicators of toxicity, based on the following equation: SI or TI (mg/g) = (weight of spleen or thymus)/body weight.

### 2.6. Bone Mineral Density (BMD)

The excised tibias were thawed and kept wrapped in gauze soaked with cold 0.9% saline solution at 4 °C for 12 h before the experiment. Then, the whole tibia BMD was measured by dual energy X-ray absorptiometry (DXA).

### 2.7. Measurement of Bone Hydroxyproline (Hyp) Content

The content of Hyp, the specific amino acid in collagen, was determined as an indicator of collagen content. Approximately 50 mg of bone tissues were powdered in a liquid nitrogen bath, hydrolyzed by sodium hydroxide and oxidized by chloramine-T. Then, the Hyp content was determined according to the introduction of the Hyp assay kit (Nanjing Jiancheng Bioengineering Inst., Nanjing, China).

### 2.8. Serum Biochemical Analysis

The alkaline phosphatase (ALP) concentration and the level of tartrate-resistant acid phosphatase-5b (TRAP-5b) activity in serum were evaluated as markers of bone formation and absorption respectively, according to the manual of commercially available ELISA kits (Nanjing Jiancheng Bio Inst., Nanjing, China).

### 2.9. Histological Analysis

The excised lumbar vertebrae L3 of mice were fixed in 10% formalin solution for 48 h, decalcified in 7.5% ethylene diaminetetraacetic acid (EDTA) in 0.1 mol/L cacodylate buffer over 1 week, dehydrated using graded ethanol and vitrified by dimethylbenzene and paraffin sectioning. Then, haematoxylin/eosin (HE) staining was performed. The changes in histology were observed by light microscopy at 100× magnification.

### 2.10. Western Blotting Analysis

Bone proteins were extracted from femurs by being lysed in lysis buffer containing protease inhibitors. Protein concentration was calculated using the BCA protein assay kit. The aliquots of samples were separated using 10% sodium dodecyl sulfate-polyacrylamide gel electrophoresis (SDS-PAGE), and transferred onto a polyvinylidene fluoride (PVDF) membrane. After blocking, the membrane was incubated (overnight at 4 °C) with the primary antibodies against TGF-β1, Smad3, Smad7, α2 and β1, respectively, followed by incubation with secondary antibody conjugated with horseradish peroxidase. An anti-glyceraldehyde-3-phosphate dehydrogenase (GAPDH) antibody was used for GAPDH evaluation as an internal control. Immunoreactive bands were visualized using chemiluminescent imaging system (Fusion SL2, Vilber Lourmat, Marne-la-Vallée Cedex, France), and the intensities of the bands were analyzed by Image Pro Plus Software 6.0.

### 2.11. Statistical Analysis

All data were expressed as mean ± standard deviation (SD). The one-way Analysis of Variance (ANOVA) with respected measurements was carried out to determine statistical differences using SPSS 17.0 (SPSS Inc., Chicago, IL, USA). The difference was considered statistically significant when *p* < 0.05.

## 3. Results

### 3.1. Characteristics of Collagen Hydrolysates (CHs)

Two kinds of collagen hydrolysates were obtained by protease hydrolysis (named ACH and CCH, respectively). As shown in Table 1, ACH and CCH had a similar molecular weight distribution, mainly ranging below 1000 Da.

As shown in Table 2, ACH and CCH showed similar amino acid compositions. Gly was the dominant amino acid in CHs, consistent with the highly repeated Gly-X-Y sequence in collagen triple helix. Besides, the gelatin was also rich in Glu, Pro, Arg, Thr and Hyp. Met, Val and Cys were the lowest amino acids of CHs.

### 3.2. Viscera Index and Body Weight

As shown in Table 3, the body weight of the YC group increased during the whole experiment. However, there were no significant differences in body weight between the M and treatment groups. Furthermore, no significant differences in SI and TI of aged mice were observed. These results indicated that CHs or proline intake at the current dose had no obvious toxicological effects on mouse growth.

### 3.3. BMD and Hyp Content

**Figure 1:**
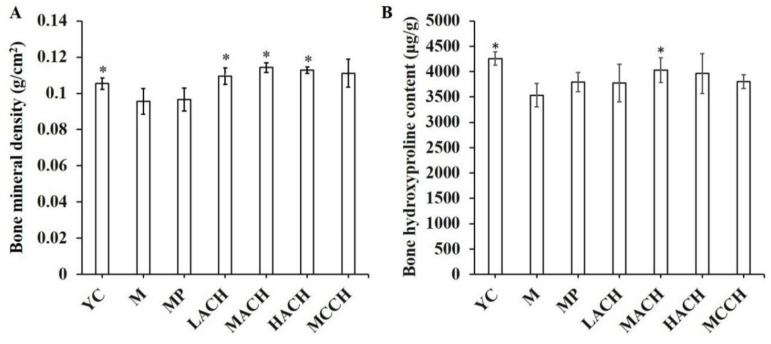
Effect of collagen hydrolysates on bone mineral density and hydroxyproline content in chronologically aged mice. (**A**) Bone mineral density; (**B**) Bone hydroxyproline content. YC, young control group; M, model group; MP, medium dose of proline; LACH, MACH and HACH represent the low, medium and high dose of ACH, respectively; MCCH, medium dose of CCH. * means a significant difference was observed compared to M group (*p <* 0.05).

Changes in BMD and Hyp content were shown in Figure 1. The BMD of the M group was significantly lower than that of the YC group (*p* < 0.05), indicating significant bone loss in chronologically aged mice. Oral administration of ACH and MCCH could significantly improve the tibia BMD, with MACH having the best effect. However, MP had no beneficial effect on the tibia BMD.

The Hyp content of the femurs from the M group was significantly lower than that of the YC group (*p* < 0.05). ACH intake could significantly increase the Hyp content, resulting in a significant difference between the MACH and M group, whereas the MCCH and MP groups had no significant improvement of femur Hyp content compared to the M group.

### 3.4. Serum Biochemical Markers

As shown in Table 4, the ALP level in the M group was significantly lower than that in the YC group (*p* < 0.05). With the treatment of CHs, the level of ALP increased and a significant difference between the MACH and M groups was observed (*p* < 0.05). MCCH and MP had no significant effect on improving the level of ALP in aged mice.

Besides, aging induced an increase in TRAP-5b activity, although there was no statistical significance (M group vs. YC group). CH intake significantly reduced the activity of TRAP-5b, with MACH possessing the best effect (*p* < 0.05). MCCH also significantly decreased the activity of TRAP-5b (*p* < 0.05), yet the effect of MP was insignificant.

### 3.5. Histological Analysis

**Figure 2:**
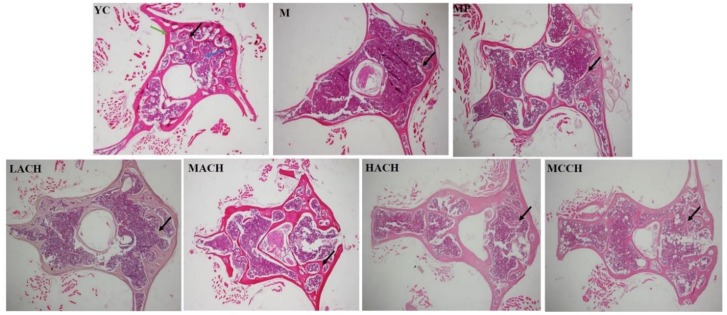
Effect of collagen hydrolysates on vertebral histology in chronologically aged mice (HE, 100×). YC, young control group; M, model group; MP, medium dose of proline; LACH, MACH and HACH represent the low, medium and high dose of ACH, respectively; MCCH, medium dose of CCH. Bony trabeculae, bone marrow and cortical bone in lumbar issue were shown as black, blue and green arrows, respectively.

As shown in Figure 2, the inner spongy bone (black arrows) in the M group showed significant architectural deterioration, with widened bone marrow spaces compared to the YC group, indicating that aging caused a serious damage of the lumbar inner structure. With the treatment of CHs, the architecture of the trabeculae was improved in terms of more branching and anastomosing of the bony trabeculae arising from the cortical bone (green arrows). MACH exerted the best effect on normalizing the integrality of bone trabeculae, and was better than MCCH at the identical dose. The effect of MP on improving the architecture of bony trabeculae is less obvious than that of CHs.

### 3.6. Effect of CHs on TGF-β/Smad Signaling Pathway

**Figure 3:**
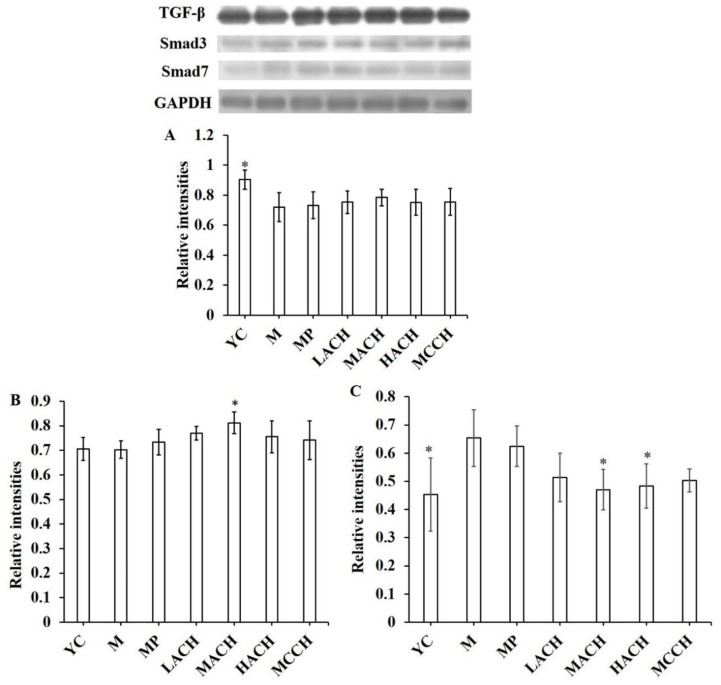
Effect of collagen hydrolysates on the transforming growth factor β (TGF-β)/Smad signaling pathway in chronologically aged mice. GAPDH, glyceraldehyde-3-phosphate dehydrogenase. (**A**) TGF-β; (**B**) Smad3; (**C**) Smad7. YC, young control group; M, model group; MP, medium dose of proline; LACH, MACH and HACH represent the low, medium and high dose of ACH, respectively; MCCH, medium dose of CCH. * means a significant difference was observed compared to M group (*p <* 0.05).

As shown in Figure 3, the expression of TGF-β in the M group was significantly decreased compared to the YC group (*p* < 0.05). There was no significant increase in the expression of TGF-β after treatment with CHs or proline. The expression of Smad3 between the YC and M groups showed no obvious differences. MACH intake significantly increased the expression of Smad3 compared to the M group (*p* < 0.05), while there were no significant differences between the other treated groups and the M group. On the contrary, CHs could decrease the level of overexpressed Smad7. MACH and HACH showed significant effects on regulating the expression of Smad7 (*p* < 0.05), whereas proline and CCH intake had no significant effect on Smad7.

### 3.7. Effect of CHs on the Expression of Integrin α2β1

**Figure 4:**
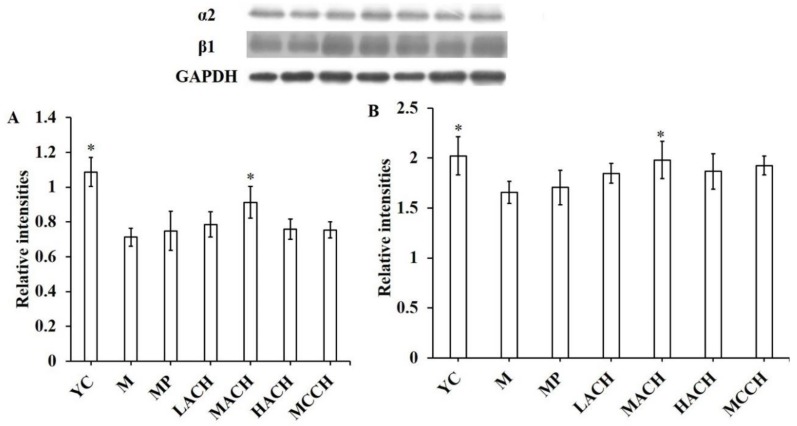
Effect of collagen hydrolysates on the expression of the α2β1 integrin receptor in chronologically aged mice. GAPDH, glyceraldehyde-3-phosphate dehydrogenase. (**A**) α2 subunit; (**B**) β1 subunit. YC, young control group; M, model group; MP, medium dose of proline; LACH, MACH and HACH represent the low, medium and high dose of ACH, respectively; MCCH, medium dose of CCH. * means a significant difference was observed compared to M group (*p <* 0.05).

The effect of CHs on the expression of integrin α2β1 is shown in Figure 4. The expression of the α2 and β1 integrin subunits in the M group was significantly decreased compared to the YC group (*p* < 0.05). After gavage with CHs, the expression of the α2 and β1 integrin subunits in each aged group showed an increasing tendency, with a significant increase in the MACH group. At the identical dose, MCCH and MP exerted a less obvious effect on increasing the expression of integrin α2β1.

## 4. Discussion

Due to an increased aging population, osteoporosis has become a major health issue demanding innovative disease management. Most of the studies have been carried out to evaluate the effects of CHs on preventing bone loss in young OVX animals [22,23,24], which are powerful and acceptable models of human postmenopausal osteoporosis. In order to study senile osteoporosis and bone loss in aged people, we employed the chronologically aged mouse model to investigate the effect of CHs on combating osteoporosis. Compared to the YC group, the M group (13-month-old) showed a significant decrease in BMD and Hyp content, as well as a serious degradation of bone microarchitecture. These results were similar to bone loss in osteoporosis, characterized by a decrease in BMD and a microarchitecture deterioration of trabecular bone [25]. Besides, chronological aging significantly reduced the expression of the TGF-β/Smad pathway and the α2β1 integrin receptor. With the oral administration of CHs, the contents of BMD and Hyp were significantly increased and the microarchitecture of lumbar trabeculae showed an obvious improvement as well. This may be due to the effect of CHs on upregulating the expression of the TGF-β/Smad pathway and stimulating the interaction between collagen and α2β1 integrin. These results indicated that 13-month-old mice could be used to study the effect of CHs on treating bone loss caused by osteoporosis.

Based on this model, we evaluated the effect of CH intake on adjusting bone mineral density (BMD). BMD is usually recognized as the standard measurement for the diagnosis of osteoporosis. In the present study, the BMD was higher in the ACH-treated groups than that in the M group. The result was consistent with the previous report in which marine CH intake increased the BMD and the bone strength of femurs in ovariectomized (OVX) mice [24]. Guillminet et al. has also shown that diets including porcine CHs significantly increased the BMD of OVX mice compared to the standard AIN-93N diet [15]. These results suggested that CHs have beneficial effects on preventing bone loss in a chronologically aged mice model.

As a dynamic metabolic system, bone is composed of approximately 70% inorganic salts (minerals) and 30% organic matrix by weight, with collagen accounting for over 90% in the organic component [16]. Although minerals play an important role in bone mass, without enough collagen, taking large dose of minerals can be wasteful, as there is an insufficient framework for minerals to attach to. Collagen is a protein that provides a framework for soft tissue which calcium adheres onto, creating a hardened framework [16]. Shuster reported that collagen loss in skin and bones with aging is the causal counterpart to bone density loss in senile osteoporosis [26]. In the present study, CH intake trended to increase the Hyp content in bone, with significant differences observed between the MCCH and M groups. Besides, the level of ALP, a well-known marker of bone formation, was also significantly increased by MCCH. In an in vitro study, it has been also demonstrated that CHs could stimulate the expression of type I collagen mRNA and its protein production [27,28], and the ALP activity was also increased in a dose-dependent manner [29], which was in agreement with the present study. This indicated that CHs might have the ability to increase bone formation, confirmed by the improved histological structure of lumbar vertebrae (Figure 2) in the present study.

The TGF-β1/Smad signaling pathway plays an important role in regulating osteoblast collagen synthesis and mineralization [30,31]. Previous research has discovered that aluminum trichloride inhibits osteoblast mineralization via the TGF-β1/Smad signaling pathway [32], which confirms the importance of the TGF-β1/Smad signaling pathway in modulating bone remodeling. In this study, CH administration did not exhibit significant effect on regulating the expression of TGF-β1, but had a significant effect on increasing the expression of Smad3 and reducing the expression of its intracellular inhibitor Smad7. Sowa et al. reported that Smad3 could enhance the expression of bone matrix proteins such as type I procollagen, osteopontin (OPN) and matrix Gla protein (MGP) in MC3T3 cells in a manner similar to that of TGF-β [33]. Moreover, Smad3 promoted ALP activity and mineralization through the improvement of collagen synthesis in vitro which differed from that of TGF-β. Therefore, we speculated that CHs might regulate bone remodeling via the important role of Smad3 in osteoblastic bone formation. Further studies are need to clarify the mechanism of bone formation via Smad3.

Several reports suggested that the interaction between type I collagen and α2β1 integrin receptors on cell membranes has a favorable effect on inducing osteoblastic differentiation of bone marrow cells [34,35]. In the present study, MACH intake significantly increased the level of α2β1 integrin as well as Hyp content. The cell experiment carried out by Mizuno and colleagues elaborated on the impact of collagen-α2β1 integrin interaction on osteoblastic differentiation showing increased ALP activity and expression [36], which was in accordance with the results from the present study. Therefore, taken together, we concluded that CH intake increases collagen synthesis via the TGF-β/Smad signaling pathway, and the higher level of collagen promotes osteoblastic differentiation of bone marrow cells (shown by biomarker ALP content) via collagen-α2β1 integrin interaction, which eventually leads to osteoblastic mineralization.

With respect to investigating the effect of CHs on improving bone quality, most of the studies focused on the effect of CHs on bone cell metabolism in bone forming cells (osteoblasts) [27,28,37]. However, the balance of bone is delicately regulated via the bone formation by osteoblasts and bone resorption by osteoclasts. Osteoblasts are responsible for synthesizing the bone extracellular matrix and regulating mineralization [27]. On the contrary, the unique function of osteoclasts is bone resorption [38]. TRAP-5b is a specific and sensitive biomarker of osteoclasts, and its concentration is recognized as a specific index of bone resorption [39]. In this study, CH intake exerted a significant effect on reducing TRAP-5b activity, indicating that CHs may also play an important role in reducing bone absorption served by osteoclasts. It has been proved that CHs can significantly inhibit osteoclast formation and activity in cell lines and in primary cultures [24,40]. However, the action mechanism of CHs on modulating osteoclasts still requires further evaluation.

It is worth noting that CCH, the collagen hydrolysates obtained by collagenase hydrolysis and with similar chemical characteristics to ACH (part data not shown), had no significant effect on regulating bone turnover. Therefore, collagenase, an important endogenous enzyme specifically acting on gelatin or natural collagen helixes [41], may not have great commercial potential for the production of collagen hydrolysates. Besides, proline, the dominant amino acid in collagen, also did not exhibit an obvious effect on improvement of bone remodeling compared to the M group. These results indicated that the repairing effect of CHs on bone loss in chronologically aged mice was likely due to the dominant Hyp-peptides in the bioactive compounds of CHs, rather than the supplement of the specific collagen amino acid component. The previous study has reported that oligopeptides are more bioactive than proteins, polypeptides and free amino acids [42], which might further explain the effect of CHs on preventing bone loss. The specific bioactive compounds responsible for repairing osteoporosis deserves an in-depth study. One limitation in the present study is that the effect of ACH at high doses (800 mg/kg body weight) on preventing bone loss was less significant than that of ACH at medium doses (400 mg/kg body weight). This may be because a small sample size was used in the preclinical trial, resulting in missing effect sizes. Alternatively, this might also be explained by high protein intake leading to higher urinary calcium excretion, which may result in accelerated bone resorption [43]. Despite of this, the present study could still evidence that ACH could be used as a dietary supplement in an appropriate dose to prevent bone loss.

## 5. Conclusions

The present study demonstrated that the ingestion of collagen hydrolysates from silver carp skin could combat osteoporosis in chronologically aged mice by increasing bone mineral density, hydroxyproline content, alkaline phosphatase (ALP) level as well as reducing tartrate-resistant acid phosphatase 5b (TRAP-5b) activity. The positive effect of ACH was more favorable than that of CCH. Proline intake had no significant influence on bone metabolism. CHs mainly activated the TGF-β1/Smad signaling pathway to stimulate collagen synthesis and strengthened the interaction between type I collagen and the α2β1 integrin receptor to improve mineralization and increase bone remodeling. In the future, more studies are needed to support that CHs increase osteoblast activity and decrease osteoclast activity.

## Figures and Tables

**Table 1:** Molecular weight distribution of collagen hydrolysates.

Sample ^1^	Molecular Weight Distribution (%)				
	<500 Da	500–1000 Da	1000–3000 Da	3000–5000 Da	>5000 Da
ACH	56.42 ± 0.000	28.62 ± 0.160	12.15 ± 0.339	2.15 ± 0.179	0.66 ± 0.000
CCH	54.47 ± 0.588	33.47 ± 0.312	11.94 ± 0.303	0.11 ± 0.027	0.01 ± 0.000

^1^ ACH, collagen hydrolysates prepared by alkaline proteinase; CCH, collagen hydrolysates prepared by collagenase. Values were shown as the mean ± SD.

**Table 2:** Amino acid compositions of gelatin from silver carp skin.

Amino Acid	Relative Content ^1,2^ (g/100g)		Amino Acid	Relative Content (g/100g)	
	ACH	CCH		ACH	CCH
Asp	2.37 ± 0.01	2.52 ± 0.03	Tyr	2.60 ± 0.03	1.51 ± 0.04
Glu	12.87 ± 0.06	12.80 ± 0.03	Val	0.62 ± 0.04	1.20 ± 0.03
Ser	3.01 ± 0.03	2.91 ± 0.04	Met	1.73 ± 0.15	0.62 ± 0.06
Gly	22.37 ± 0.19	22.57 ± 0.28	Cys	0.08 ± 0.00	1.05 ± 0.03
His	3.23 ± 0.06	3.38 ± 0.02	Ile	2.03 ± 0.08	2.01 ± 0.20
Thr	8.95 ± 0.06	8.89 ± 0.01	Leu	1.80 ± 0.06	1.68 ± 0.03
Ala	5.95 ± 0.42	5.84 ± 0.05	Phe	3.53 ± 0.42	3.43 ± 0.08
Pro	10.29 ± 0.62	10.57 ± 0.07	Lys	3.76 ± 0.16	3.94 ± 0.08
Arg	8.56 ± 0.16	8.44 ± 0.39	Hyp	6.26 ± 0.25	6.63 ± 0.12

^1^ The content of individual amino acids was expressed as g per 100 g of the total amino acids in gelatin from silver carp skin. ^2^ ACH, collagen hydrolysates prepared by alkaline proteinase; CCH, collagen hydrolysates prepared by collagenase. Values were shown as the mean ± SD.

**Table 3:** Body weight, spleen index (SI) and thymus index (TI) of mice.

Group ^1^	Body Weight (g)					SI ^2^ (mg/g·bw)	TI ^2^ (mg/g·bw)
	Week 0	Week 2	Week 4	Week 6	Week 8		
YC	28.28 ± 0.73 *	30.27 ± 1.62 *	31.71 ± 1.30 *	32.63 ± 1.91 *	32.70 ± 1.60 *	3.48 ± 0.69	1.82 ± 0.38
M	45.72 ± 5.51	45.55 ± 4.67	46.33 ± 5.76	46.52 ± 3.58	46.33 ± 4.01	3.83 ± 1.14	1.55 ± 0.53
MP	47.24 ± 5.40	46.96 ± 6.14	46.32 ± 5.86	47.65 ± 6.22	47.44 ± 6.00	3.64 ± 1.04	2.02 ± 0.87
LACH	46.21 ± 5.78	45.36 ± 3.92	44.65 ± 3.94	45.46 ± 3.38	45.56 ± 4.52	4.07 ± 1.01	1.31 ± 0.80
MACH	46.33 ± 5.62	44.92 ± 3.57	44.48 ± 2.64	46.22 ± 3.68	45.48 ± 2.27	4.22 ± 0.63	1.85 ± 0.97
HACH	46.72 ± 5.20	45.90 ± 5.27	46.67 ± 5.71	47.63 ± 5.60	46.84 ± 5.24	3.4 ± 0.73	2.01 ± 0.81
MCCH	46.88 ± 5.22	46.36 ± 5.36	45.13 ± 5.53	46.45 ± 5.25	45.81 ± 5.39	3.51 ± 0.92	1.45 ± 0.48

^1^, YC, young control group; M, model group; MP, medium dose of proline; LACH, MACH and HACH represent the low, medium and high dose of ACH, respectively; MCCH, medium dose of CCH. ^2^, Indicated values at week 8. All the values were shown as the mean ± SD. * means a significant difference was observed compared to M group (*p <* 0.05).

**Table 4:** Effects of collagen hydrolysates on bone serum biomarkers of mice.

Group ^1^	Dose (mg/kg·bw)	ALP Content (ng/mL)	TRAP-5b Activity (U/L)
YC	-	15.00 ± 1.33 *	44.27 ± 4.28
M	-	8.12 ± 1.50	52.16 ± 4.21
MP	400	7.81 ± 0.60	49.77 ± 4.97
LACH	200	8.38 ± 1.21	45.36 ± 2.06
MACH	400	10.67 ± 0.52 *	40.00 ± 1.35 *
HACH	800	9.16 ± 0.65	41.09 ± 1.44 *
MCCH	400	8.46 ± 1.23	42.02 ± 0.96 *

^1^, YC, young control group; M, model group; MP, medium dose of proline; LACH, MACH and HACH represent the low, medium and high dose of ACH, respectively; MCCH, medium dose of CCH. All the values were shown as the mean ± SD. * means a significant difference was observed compared to M group (p < 0.05). ALP, alkaline phosphatase; TRAP-5b, tartrate-resistant acid phosphatase-5b.
